# Risk Factors for Acute *Toxoplasma gondii* Diseases in Taiwan: A Population-Based Case-Control Study

**DOI:** 10.1371/journal.pone.0090880

**Published:** 2014-03-07

**Authors:** Ting-Yi Chiang, Ming-Chu Kuo, Chang-Hsun Chen, Jyh-Yuan Yang, Cheng-Feng Kao, Dar-Der Ji, Chi-Tai Fang

**Affiliations:** 1 Center for Research, Diagnostics and Vaccine Development, Centers for Disease Control, Ministry of Health and Welfare, Taipei, Taiwan; 2 Institute of Epidemiology and Preventive Medicine, College of Public Health, National Taiwan University, Taipei, Taiwan; 3 Division of HIV/AIDS and TB, Centers for Disease Control, Ministry of Health and Welfare, Taipei, Taiwan; 4 Department of Tropical Medicine, National Yang-Ming University, Taipei, Taiwan; 5 Department of Internal Medicine, National Taiwan University Hospital, Taipei, Taiwan; 6 Ministry of Health and Welfare and National Taiwan University Infectious Diseases Research and Education Center, Taipei, Taiwan; University of Aberdeen, United Kingdom

## Abstract

Although human toxoplasmosis is a notifiable disease in Taiwan since 2007, little is known about its risk factors. This study aimed to investigate the risk factors for acute *Toxoplasma gondii* diseases in Taiwan. We conducted a nationwide population-based case-control study. Cases of acute human toxoplasmosis notified to the Taiwan Centers for Diseases Control (Taipei, Taiwan) during 2008–2013 were compared with controls that were randomly selected from healthy *T. gondii*-seronegative blood donors who participated in a nationwide *T. gondii* seroepidemiologic study during 2009–2010. Cases and controls were matched according to age, gender and residency at an 1∶8 ratio. Structured questionnaires were used to gather information regarding risk factors. A total of 30 laboratory-confirmed acute *T. gondii* disease cases and 224 controls were enrolled. The most common clinical manifestation of the cases was flu-like symptoms (n = 20), followed by central nervous system disease (n = 4), ocular diseases (n = 3), abortion (n = 2), and congenital infection (n = 1). Multivariate conditional logistic regression showed that raw clam consumption (adjusted odds ratio [OR] = 3.7; 95% confidence interval [CI] = 1.4–9.9) and having a cat in the household (adjusted OR = 2.9; 95% CI = 1.1–7.9) were two independent risk factors for acute *T. gondii* disease. We conclude that raw shellfish consumption and domestic cat exposure were risk factors for acquiring acute *T. gondii* diseases in Taiwan. This finding may guide future research and control policies.

## Introduction

*Toxoplasma gondii*, a zoonotic protozoan pathogen, infects nearly one-third of the world’s human population [1]–[3]. Human infections can occur through three major transmission routes: food borne (consumption of meat infected by tissue cysts), animal-to-human (ingestion of oocysts shed in the feces of infected cats), and mother-to-fetus (congenital infection through the placenta during pregnancy) [2], [4]. *T. gondii* can also be transmitted through blood transfusion or organ transplantation from infected donors [5]–[7].

Acute toxoplasmosis in immunocompetent persons is usually self-limited [2], [4]. Flu-like symptoms or lymphadenopathy are the most common clinical manifestations [2], [4]. Ocular disease (chorioretinitis) with impaired vision can occur in immunocompetent persons [2], [4], either sporadically or in the context of an outbreak [8]. Congenital toxoplasmosis can cause intracranial and ocular lesions in newborns, which may lead to subsequent mental retardation or blindness [9]. For immunocompromised patients, toxoplasmosis is a life-threatening disease [2], [4]. Encephalitis is the most common clinical manifestation, but chorioretinitis, pneumonia, or multi-organ involvements can also be observed [2], [4]. The *T. gondii* disease burden [9] and its potential to cause outbreaks [8], [10] highlight the importance of identifying the risk factors related to this neglected disease.

Toxoplasmosis became a notifiable communicable disease in Taiwan in 2007. To survey the seroprevalence of *T. gondii* infection, we conducted the first country-wide cross-sectional seroepidemiologic study among Taiwanese healthy blood donors, which included a large 1,783-subject sample size, from 2009 to 2010 [11]. We found that *T. gondii* infection exists in all major regions in Taiwan, with an overall 9.3% seroprevalence. To further investigate the risk factors of human *T. gondii* diseases, we conducted a population-based matched case-control study.

## Materials and Methods

### Study Design

This was a nationwide population-based matched case-control study. Laboratory-confirmed cases of acute human toxoplasmosis reported to the Taiwan Centers for Diseases Control (CDC) (Taipei, Taiwan) during 2008–2013 were compared with controls randomly selected from the *T. gondii*-seronegative healthy blood donors who participated in our previous nationwide seroepidemiologic study, which occurred during 2009–2010 [11]. Cases and controls were matched according to age, gender and residency.

### Ethical Statement

The study procedure was approved by the institutional review board of National Taiwan University Hospital (IRB number 201204055RIC). Written informed consent was obtained from all participants.

### Setting

Starting in 2007, all cases with either clinical illnesses or laboratory findings compatible with acute *T. gondii* diseases were required to be reported to the Taiwan CDC. The clinical illnesses included 1) probable congenital infections in newborn infants, including intracranial calcification, hydrocephalus, microcephaly, chorioretinitis, glaucoma, pneumonia, myocarditis, skin rash, hepatosplenomegaly, or neonatal jaundince, and 2) probable acquired infections, including lymphadenopathy or flu-like symptoms in immunocompetent persons, as well as chorioretinitis, pneumonia, pericarditis, myocarditis, or encephalitis in immmunocompromised patients. The laboratory findings included histopathology, serology, nucleic acid detection, or isolation-identification [12].

Physicians were required to send whole blood, paired serum, cerebrospinal fluid, amniotic fluid, or other relevant samples of reported patients to Taiwan CDC to obtain laboratory confirmation of the diagnoses.

### Case Definition

The reported acute toxoplasmosis cases were considered laboratory-confirmed when one of the following two criteria were met: 1) serologic assays indicated an acute infection or 2) *T. gondii* DNA was detected in the peripheral blood/body fluids by real-time polymerase chain reaction (PCR).

### Serological Diagnosis

Paired serum samples taken 14–21 days apart were tested using commercial enzyme immunoassays (bioMérieux, Marcy l’Etoile, France) with an automated Vitek Immuno Diagnostic Assay System (VIDAS). The analyses were performed as instructed by the manufacturers. For the IgG and IgM assays, positive results were defined as values of ≥8 international units (IU)/ml and index values of ≥0.65. Equivocal results ranged from 4 to 8 IU/ml, and index values ranged from 0.55 to 0.65. Negative results were defined as <4 IU/ml and index values of <0.55.

If the analyses of the paired serum samples revealed a significant increase in the IgG antibody titers or positive results from both IgM and IgG assays, then we performed a subsequent *T. gondii* IgG avidity assay (bioMérieux, Marcy l’Etoile, France). The results were interpreted in accordance with the manufacturer’s instructions. A high avidity test result excluded a recently acquired infection within 4 months of serum sampling. Low IgG avidity was defined as an index value <0.200, equivocal IgG avidity was defined as 0.200≤ index <0.300, and high IgG avidity was defined as an index value ≥0.3.

### Real-time PCR Diagnoses

DNA from peripheral blood and/or body fluids (cerebrospinal fluid or amniotic fluid) was extracted using the QIAamp DNA Mini Kit (QIAGEN, Valencia, CA, USA), according to the manufacturer’s protocol. We performed a real-time PCR assay targeting the 529-bp repeat element (RE) of *T. gondii* as previously described [13], with some modifications. The cycling parameters were as follows: preheat to 95°C for 10 min, followed by 45 cycles of 95°C for 15 seconds, 55°C for 30 seconds, and 72°C for 15 seconds. Amplification was performed using the CFX96 real-time PCR detection system (Bio-Rad Laboratories, Hercules, CA, USA). The final reaction mix contained 12.5 µl 2× KAPA PROBE FAST qPCR Master Mix (KAPA Biosystems, Boston, MA, USA), 400 nM of each primer, 800 nM of TaqMan probe, and 5 µl of template DNA in a 25 µl reaction volume. A sample was considered to be positive if the cycle threshold (Ct) value was <40.

### Control Selection

Controls were randomly selected from *T. gondii*-seronegative healthy blood donors who participated in our previous nationwide seroepidemiologic study, which occurred between 2009 and 2010 [11]. Random sampling was conducted using a random number generator. We aimed to select eight control subjects for each case, which were individually matched by age group (16–25, 26–35, 36–45, 46–55, >55), gender and residency (six geographical regions: northern, northwestern, central, southwestern, southern, and eastern Taiwan) [11].

### Study Questionnaire

We used the same questionnaire to collect basic demographic data and information on possible risk factors for all case patients and control subjects. The basic demographic data included age, gender, education, residence, and occupation. Risk factor data included drinking water sources (tap water, spring water, groundwater, and bottled water), the consumption of raw/undercooked fish, clams, mussels, oysters, lamb, beef and pork, the consumption of raw vegetables, contact with animals (cats and dogs), gardening or agriculture, prior blood transfusions, and whether patients had previously lived abroad. Informed consent and questionnaires were mailed to the case patients to request their participation.

### Information on HIV Infection

Both human immunodeficiency virus (HIV) infection and acquired immunodeficiency syndrome (AIDS) have been reportable diseases in Taiwan since 1984. The national HIV/AIDS registry was used to ascertain whether the acute toxoplasmosis case patient is also a notified HIV/AIDS case. Case patients who are not notified HIV/AIDS cases were contacted to obtain their written informed consent to receive an HIV test using the Serodia-HIV test kit (Fujirebio, Tokyo, Japan), a particle-agglutination test for detecting HIV type 1 and/or type 2 antibodies. All control subjects voluntarily received routine HIV testing with enzyme-linked immunosorbent assays (ELISA) at the time of blood donation, as previously described [11].

### Statistical Analyses

All data analyses were performed using SAS, version 9.2 for Windows (SAS Institute Inc., Cary, NC, USA). The risk factors were analyzed using conditional logistic regression. For multivariate analyses, all potential risk factors were included in the maximum model. We then performed stepwise regression procedures. All analyses were two-tailed, and p<0.05 was considered statistically significant.

## Results

### Cases

**Figure 1:**
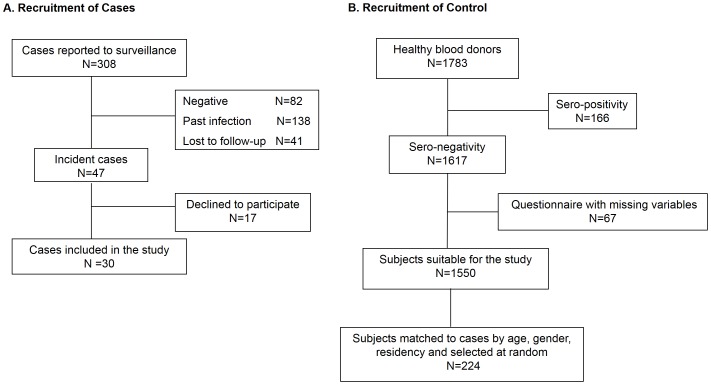
Summary of the recruitment process, which included (A) case and (B) control subject recruitment.

From January 2008 through March 2013, a total of 308 acute human toxoplasmosis cases were reported, and 47 of them were laboratory-confirmed. The remaining 261 cases included subjects with past infections (positive IgG assay results but with a high avidity, n = 138), negative serological and real-time PCR results (n = 82), or those who were lost to follow-up (lack of the second serum sample, n = 41) (Figure 1A).

Of the 47 laboratory-confirmed cases, 40 were seropositive for both anti-*T. gondii* IgM and IgG in the paired serum samples and had a low IgG-avidity compatible with a recently acquired infection within 4 months. The paired anti-*T. gondii* IgG positive sample titers ranged from 67 to 2100 IU/ml (median titers: 404.5 IU/ml) and 105 to 2460 IU/ml (median titers: 468.0 IU/ml), respectively. The remaining seven cases were diagnosed by *T. gondii*-specific DNA detection in the peripheral blood and/or the cerebrospinal fluid.

All 47 patients with laboratory-confirmed acute *T. gondii* diseases were invited to join the present study, and 30 of them agreed to participate and completed the study questionnaire. The basic demographic characteristics of the 30 cases are shown in Table 1. There were no statistically significant differences in the number of participating case patients per 100,000 people across the six geographic regions (p = 0.508, chi-square test) (Table 1).

**Table 1:** Demographic characteristics of cases and controls.

	Cases (N = 30)	Controls (N = 224)
Characteristics	n (%)	n (%)
Residency (Regional population†)		
Northern Taiwan (6,910,467)	7 (23.3)	58 (25.0)
Northwestern Taiwan (3,487,664)	5 (16.7)	36 (16.1)
Central Taiwan (4,485,041)	9 (30.0)	69 (30.8)
Southwestern Taiwan (3,408,726)	5 (16.7)	34 (15.2)
Southern Taiwan (3,742,589)	2 (6.7)	16 (7.1)
Eastern Taiwan (1,028,692)	2 (6.7)	13 (5.8)
Gender		
Male	13 (43.3)	104 (46.4)
Female	17 (56.7)	120 (53.6)
Age		
16–25	5 (16.7)	40 (17.9)
26–35	13 (43.3)	100 (44.6)
36–45	5 (16.7)	37 (16.5)
46–55	2 (6.7)	15 (6.7)
>55	5 (16.7)	32 (14.3)
Childbearing-aged (16–45 y/o) woman	11 (36.7)	81 (36.2)
Blood group type		
O	16 (53.6)	94 (41.3)
A	7 (21.4)	65 (29.8)
B	5 (17.9)	43 (19.7)
AB	2 (7.1)	22 (9.1)
Educational level		
High school and below	16 (53.6)	81 (35.1)
College and above	14 (46.4)	143 (64.9)
Occupation		
Laborer	7 (23.3)	41 (18.3)
Businessman/employee	17 (56.7)	125 (55.8)
Student/unemployed	6 (20.0)	58 (25.9)

† Average of end-year population, 2008–2012.

### Controls

During 2009–2010, a total of 1,783 healthy blood donors participated in our nationwide seroepidemiologic survey, and 1,550 of them were *T. gondii*-seronegative and had completed the study questionnaire. From these 1,550 subjects, we randomly selected 224 (individually matched to cases by age, gender and residency) as the controls for this study (Figure 1B). The control numbers per case were eight (24 pairs), seven (1 pair), six (2 pairs), five (2 pairs), and three (1 pair).

The demographic characteristics of the controls are shown in Table 1. The 224 controls had an age distribution similar to the 30 case patients (mean: 36.0 years [range: 18 to 65 years] vs. mean: 36.5 years [range 16 to 60 years]). The gender and residency distributions were also similar between the two groups (Table 1).

### Clinical Manifestations of Acute *T. gondii* Diseases

The clinical manifestations and laboratory diagnosis of acute toxoplasmosis in the 30 case patients are shown in detail in Table 2. The most common clinical manifestations were flu-like symptoms (n = 20), followed by central nervous system disease (n = 4), ocular diseases (n = 3), abortion (n = 2), and a congenital infection (n = 1) (Table 2).

**Table 2:** Clinical presentations and laboratory diagnoses of 30 acute *Toxoplasma gondii* diseases cases.

	n (%)
**Clinical presentation**	
Flu-like symptoms	20 (66.7)
Lymphadenopathy	20 (66.7)
Fever	3 (10.0)
Chest tightness	1 (3.3)§
Ocular diseases¶	3 (10.0)
Central nervous system diseases*	4 (13.3)
Congenital infection	1 (3.3)
Other (previous abortion)	2 (6.7)
**Laboratory diagnosis**	
Positive IgM and low IgG-avidity	25 (83.3)
PCR†	5 (16.7)
Blood	5 (16.7)
CSF	2 (6.7)

† A total of 5 tested cases were PCR positive, and among them, 2 cases.

were identified with *T. gondii-*specific DNA both in the peripheral blood and the cerebrospinal fluid.

*Of the 4 central nervous system disease cases, 3 were HIV-infected patients and one refused to receive HIV testing.

¶ Of the 3 ocular disease cases, 1 was an HIV-infected patient, 1 was an HIV–negative patient, and one refused to have an HIV examination.

§ An HIV-infected patient developed flu-like symptoms.

Five of the 30 case patients had reported HIV infection, and all of them had fulfilled the clinical AIDS criteria before (n = 4) or at the time (n = 1) of acute toxoplasmosis onset. Three of the subjects presented with central nervous system diseases (encephalitis), one presented with ocular diseases (chorioretinitis), and one presented with flu-like symptoms (Table 2, footnote).

Twelve of the remaining 25 case patients agreed to receive HIV testing. The HIV testing results were negative for all 12 case patients. Compared with these 12 HIV-negative case patients, the 5 HIV-infected case patients were significantly more likely to present with central nervous system diseases (3/5 [60%] vs. 0/12 [0%], p = 0.015, Fisher’s exact test). The probability to present with ocular diseases was not significantly different between the 5 HIV-infected and the 12 HIV-negative case patients (1/5 [20%] vs. 1/12 [8%], p = 0.515, Fisher’s exact test).

### Risk Factors for Acute *Toxoplasma gondii* Diseases

In the univariate analysis, three variables were found to be significantly associated with acute *T. gondii* diseases: raw clam consumption (crude odds ratio [OR] = 3.6; 95% CI: 1.4–9.3, p = 0.008), having a cat in the household (crude OR = 2.8; 95% CI: 1.1–7.3, p = 0.040), and HIV infection (crude OR = 53.8; 95% CI: 7.3–infinity, p<0.0001) (Table 3). Multivariate analyses, with stepwise regression, demonstrated that raw clam consumption (adjusted OR = 3.7; 95% CI: 1.4–9.9, p = 0.008) and having a cat in the household (adjusted OR = 2.9; 95% CI: 1.1–7.9, p = 0.03) were independent risk factors for acute *T. gondii* diseases (Table 4).

**Table 3:** Univariate analysis of the variables associated with *Toxoplasma gondii* diseases.

	Case patients	Control subjects	Crude Odds Ratio	
Variable	(N = 30)	(N = 224)	(95% Confidence interval)	p-value
Educational level				
High school and below	16	81	2.2 (0.9–5.1)	0.079
College and above	14	143	1.0	
Blood group type				
O	16	94	1.0	0.744
A	7	65	0.6 (0.2–1.7)	
B	5	43	0.7 (0.2–2.1)	
AB	2	22	0.6 (0.1–2.7)	
Valley water consumption				
Yes	3	20	1.2 (0.3–5.1)	0.769
No	27	204	1.0	
Undercooked beef consumption				
Yes	12	62	1.8 (0.8–3.9)	0.149
No	18	162	1.0	
Undercooked lamb meat consumption				
Yes	2	8	1.9 (0.4–9.7)	0.397
No	28	216	1.0	
Undercooked pork meat consumption				
Yes	1	8	0.9 (0.1–7.4)	0.943
No	29	216	1.0	
Raw fish consumption				
Yes	22	152	1.4 (0.6–3.5)	0.418
No	8	72	1.0	
Raw oyster consumption				
Yes	10	58	1.5 (0.6–3.4)	0.349
No	20	166	1.0	
Raw clam consumption				
Yes	7	15	3.6 (1.4–9.3)	0.008
No	23	209	1.0	
Uncooked vegetables consumption				
Yes	23	164	1.5 (0.5–3.9)	0.459
No	7	60	1.0	
Cats in the neighborhood				
Yes	21	136	1.6 (0.7–3.7)	0.620
No	9	88	1.0	
Cat in the household				
Yes	8	30	2.8 (1.1–7.3)	0.040
No	22	194	1.0	
Gardening or agriculture				
Yes	15	75	2.2 (0.97–5.1)	0.058
No	15	149	1.0	
Blood transfusion				
Yes	5	21	1.96 (0.7–5.8)	0.224
No	25	203	1.0	
Travel				
Yes	8	36	2.1 (0.8–5.5)	0.110
No	22	188	1.0	
Living abroad (>3 months)				
Yes	1	9	1.0	0.843
No	29	215	1.2 (0.2–10.2)	
HIV-positive†				
Yes	5	0	53.8 (7.3–infinity)§	<0.0001§
No	12	131	1.0	

† Only 17 cases had a known HIV status.

§ Odds ratios and p values were computed using exact conditional logistic regression due to sparse data.

**Table 4:** Multivariate logistic regression for risk factors of acute *Toxoplasma gondii* diseases.

Variable	Adjusted odds ratio (95% Confidence interval)	p-value
Raw clam consumption	3.7 (1.4–9.9)	0.008
Cat in the household	2.9 (1.1–7.9)	0.03

## Discussion

The present study is the first nationwide population-based case-control study that assessed the risk factors of acute *T. gondii* diseases in Taiwan. In addition to the countrywide coverage of case patients and matched controls, the present study had methodological strength because it used serological assays and real-time PCR to confirm the diagnosis of recently acquired infection in all case patients and to confirm the seronegative status in all of the control subjects. The results indicated that household cat exposure and raw clam consumption were two independent risk factors for acquiring acute *T. gondii* diseases in Taiwan. These findings may guide future research and control policies.

Cats are a definite *T. gondii* host [14]–[15]. It has been well documented that *T. gondii* oocysts, which are shed in infected cat feces, are a major source of human infection [15]. Cat exposure has been consistently demonstrated to be a predictive *Toxoplasma* seropositivity factor in prior seroepidemiologic studies [11], [16]–[22]. A nationwide case-control study conducted by the Centers for Diseases Control and Prevention (Atlanta, GA, USA) identified that kitten exposure is a *Toxoplasma* infection risk factor in the United States [23]. In keeping with the existing literature, the present study found that household cat exposure is a risk factor for acute *T. gondii* diseases in Taiwan. Our previous survey found that women of childbearing age are more likely to raise cats than older women in Taiwan [11]. Health education targeted at women of childbearing age may be required to minimize the health risks associated with domestic cat-raising.

Farmed shellfish can concentrate *T. gondii* oocysts from contaminated water through their water-filtering feeding behaviors [24]. Uncooked contaminated shellfish consumption has been proposed as a potential human infection source. A nationwide case-control study conducted in the United States showed that eating raw oysters, clams, or mussels is a risk factor for *Toxoplasma* infection [23]. In our previous seroepidemiologic survey [11], we found that raw shellfish consumption is a predictive factor for *Toxoplasma* seropositivity. The present study further demonstrated that raw clam consumption is also a risk factor for acute *T. gondii* diseases in Taiwan. More investigations on the health risks of eating raw shellfish are required. Future directions include the detection of *T. gondii* DNA in edible shellfish and the identification of the contamination source in farmed shellfish.

Toxoplasmosis of the brain is a well-known AIDS-defining illness for HIV-infected patients [25]. The impaired cell-mediated immunity associated with HIV infection is likely to predispose these patients to life-threatening *T. gondii* infection and should therefore be a risk factor for acquiring acute toxoplasmosis. In the present study, we observed that HIV-infected case patients were significantly more likely to develop life-threatening central nervous system diseases compared with HIV-negative case patients. Our findings thus provide epidemiologic evidence to support the important role of impaired cell-mediated immunity in pathogenesis of toxoplasma encephalitis. Our data also demonstrated that HIV infection was a significant risk factor for acquiring acute *T. gondii* diseases in the univariate analysis. However, 13 patients, including one with encephalitis and one with chorioretiinitis, declined to receive HIV testing. The small sample size of case patients with a known HIV status (n = 17) limited the statistical power of the multivariate regression analyses in examining the role of HIV infection as a risk factor for acute *T. gondii* diseases.

It appears that risk factors for acute toxoplasmosis can vary across different geographic regions. A European multicenter case-control study of 252 pregnant women with acute infections and 858 control subjects was conducted in several regions of France, Denmark, Italy, Belgium and Sweden. Eating undercooked meats, contact with soil, and travel outside Europe or North America were significantly associated with acute toxoplasmosis [26]. Moreover, a case-control study of 148 recently infected adults and 413 controls in the United States showed that eating ground beef, rare lamb, and other undercooked meats, as well as drinking unpasteurized goat milk, having more than 3 kittens, and eating raw oysters, clams, and mussels were risk factors for human *Toxoplasma* infection [23]. Undercooked meat consumption was also associated with *Toxoplasma* seroconversion or seropositivity in case-control studies of pregnant women in France and Italy [16], [27]. In all of the above studies, undercooked meat consumption was a strong risk factor; however, it was not shown to be a risk factor in our study. The probable reason is that the majority of Taiwanese people prefer well-cooked food, and the number of cases attributable to raw meat in the present study was thus too small to be examined with sufficient statistical power.

Similar to other observational studies, our study had several limitations. First, as stated above, the limited study size of our case-control study may have restricted its ability to detect some risk factors. For example, it is likely that HIV infection is an independent risk factor for acute *T. gondii* disease onset; however, this could not be shown in the multivariate analyses due to the very small number of case patients with a known HIV status. Second, previous studies in Taiwan demonstrated that aboriginal people, who constitute a significant proportion of the population in eastern Taiwan, have a much higher *Toxoplasma* seroprevalence compared to non-aboriginal people [11], [28]–[29]. The use of residency-matched controls in the present study eliminates the confounding effect from the ethnic factors. The limited participating rate among the case patients (30/47, 64%), nevertheless, precludes a conclusive analysis on the effect of residency in eastern Taiwan (a surrogate marker for aboriginal ethnicity) on the risk for acute *T. gondii* diseases. Third, the case enrollment period was from 2008 to 2013, which was slightly different from the control subject enrollment period (2009 to 2010). Nevertheless, the potential bias is most likely minimal because there were no known life style changes, such as eating and pet raising habits, during the study period.

In conclusion, the present study identified that the major risk factors associated with acute *T. gondii* diseases were domestic cat exposure and raw clam consumption in Taiwan. Women of childbearing age [30] and immunocompromised patients, especially those with HIV infection, should be counseled to avoid these two risk factors. This information should be provided as public health education in an effort to prevent human toxoplasmosis.
